# Coin pica‐induced gastric perforation resulting from ingestion of 1,894 coins, 8 kg in total: case report and review of published works

**DOI:** 10.1002/ams2.318

**Published:** 2017-10-24

**Authors:** Kosuke Sekiya, Shusuke Mori, Yasuhiro Otomo

**Affiliations:** ^1^ Trauma and Acute Critical Care Medical Center Tokyo Medical and Dental University Tokyo Japan

**Keywords:** Coin pica, gastric perforation, metal toxicity, psychiatric disorders

## Abstract

**Case:**

Pica is common among patients with psychiatric disorders, but only a few cases regarding coin pica have been reported. A 51‐year‐old man with depression complaining of fatigue was found to have numerous coins in the esophagus and the stomach on X‐rays. He had a peritoneal sign and underwent an emergency laparotomy.

**Outcome:**

The surgical findings showed perforation on the anterior wall of the gastric body and coins in the stomach, which were removed manually, followed by an omental patch. Residual coins in the esophagus were removed by endoscopy. The coins totaled 1,894, weighing 8,076 grams. The patient was then diagnosed as schizophrenic. He was asymptomatic for metal toxicity and was finally transferred to a psychiatric hospital.

**Conclusion:**

This pica case is the first to show coin pica can lead to gastric perforation, and also reports the largest amounts of coins ingested by a person to date.

## Introduction

Pica is common among patients with psychiatric disorders.^1^ Although there are various kinds of objects or materials for ingestion in pica cases, reports regarding ingestion of a large number of coins are rarely seen.^2^, ^3^, ^4^, ^5^, ^6^, ^7^ Coin ingestion, regardless of incidentally or accidentally swallowed, is also a common disease occasionally seen in emergency departments; however, these cases are usually marked by small numbers of coins and, therefore, few symptoms are reported, if any. Nonetheless, there are cases in which patients confess to purposely ingesting coins, leading to complications such as narrowing of the gastrointestinal tract or lumen obstruction. Ingesting a large number of coins can only be deemed intentional, and hence, it is usually related to a psychiatric disorder. Pica sometimes causes gastrointestinal tract perforation.^8^ Foreign bodies such as batteries, sharp objects, and metal materials are the main cause of perforation seen in pica patients. Ingestion of substantial amounts of any foreign body may also lead to perforation. Coin pica has rarely been reported. We herein report this unique case of pica leading to gastric perforation due to ingestion of the largest number of coins ever recorded.

## Case

**Figure 1:**
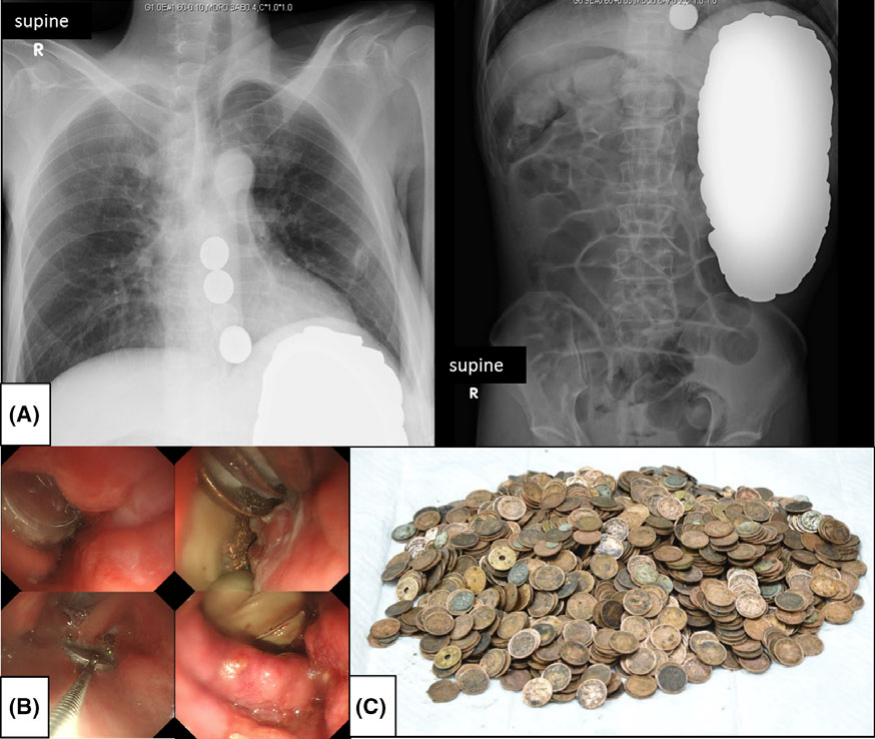
Coin pica‐induced gastric perforation in a 51‐year‐old man with schizophrenia who ingested 1,894 coins. A, Plain chest and abdominal X‐rays showed numerous round‐shaped radiopaque foreign bodies suggestive of coins. B, Endoscopic removal of the coins in the esophagus. C, All the coins removed from the digestive tract.

A non‐medicated 51‐year‐old man with a 30‐year history of depression presented to a local hospital complaining of fatigue and loss of appetite. The initial vital signs were: Glasgow Coma Scale 15 (E4V5M6); blood pressure, 139/81 mmHg; heart rate, 114 b.p.m.; respiratory rate, 31 breaths/min; body temperature, 37.2°C; and SpO_2_, 98%. Physical examination revealed that his abdomen was hard and tender on the left flank, showing signs of peritoneal irritation. The blood test revealed leukocytosis, iron deficiency anemia, thrombocytosis, hypoalbuminemia, hyponatremia, and high C‐reactive protein, but did not show liver or kidney dysfunction (Table 1). Plain chest and abdominal X‐rays showed numerous round‐shaped radiopaque foreign bodies suggestive of coins (Fig. 1A). The patient was diagnosed with coin pica complicated with gastrointestinal tract perforation and he was transferred to the hospital for surgical treatment. On the same day, an emergency laparotomy was performed. Following celiotomy, a hole approximately 5 mm in size was observed on the anterior wall and greater curvature side of the stomach, from which coins were identified. The stomach was remarkably distended with a large number of coins. No coins spilled out from the stomach to the abdominal cavity. The hole seemed to be eroded by the pressure of heavy solid materials. In order to remove the coins, an incision of approximately 5 cm was made on the greater curvature of the stomach separately from the hole that was first observed. After coins in the stomach were removed by hand, the incision was closed primarily. The hole and surrounding tissue seemed structurally weak, presumably due to erosive changes, and, therefore, was closed by a single stitch with the same suture material and an omental patch was applied to the site for reinforcement.

**Table 1:** Laboratory data at initial presentation of a 51‐year‐old man with schizophrenia who ingested 1,894 coins

WBC	22,000/mm^3^	Alb	2.1 g/dL	LDH	167 U/L
RBC	440 × 10^4^/mm^3^	AST	20 U/L	CPK	192 U/L
Hg	11.3 g/dL	ALT	13 U/L	Amy	34 U/L
Ht	34.20%	T‐Bil	1.2 mg/dL	CRP	36.0 mg/dL
MCV	77.7 fL	BUN	18 mg/dL		
MCH	25.7 pg	Cre	0.69 mg/dL		
MCHC	33.00%	Na^+^	130 mEq/L		
Plt	71.7 × 10^4^/mm^3^	K^+^	3.9 mEq/L		

Alb, albumin; ALT, alanine aminotransferase; Amy, amylase; AST, aspartate aminotransferase; BUN, blood urea nitrogen; CPK, creatine phosphokinase; Cre, creatinine; CRP, C‐reactive protein; Hg, hemoglobin; Ht, hematocrit; LDH, lactate dehydrogenase; MCH, mean corpuscular hemoglobin; MCHC, mean corpuscular hemoglobin concentration; MCV, mean volume; Plt, platelets; RBC, red blood cells; T‐Bil, total bilirubin; WBC, white blood cells.

Subsequently, a small number of residual coins in the esophagus were removed by endoscopy (Fig. 1B). The total number of coins removed was 1,894 and their weight was 8,076 g. All of the ingested coins were Japanese yen consisting of 140 1‐yen coins, 99 5‐yen coins, 1,642 10‐yen coins, 8 50‐yen coins, and 5 100‐yen coins (Fig. 1C). The postoperative course was uncomplicated. The patient underwent a psychiatric evaluation and was diagnosed with schizophrenia. He was transferred to a psychiatric facility for treatment on post‐operative day 16. The laboratory results of serum concentration of copper and zinc were 56 μg/dL (reference value, 80–135) and 21 μg/dL (reference value, 80–130), respectively.

## Discussion

Pica is characterized by the persistent eating of non‐nutritive substances, and is mainly seen in psychiatric patients, children, young adolescents, and pregnant women.^1^, ^9^ Lethal complications of pica include intestinal obstruction and perforation with peritonitis.^8^ Other findings at autopsy may include airway obstruction and heavy metal poisoning.^9^ Presenting symptoms and signs of such complications may be subtle or masked given the nature of underlying conditions. Therefore a careful evaluation of the medical histories of individuals with pica is necessary to provide pertinent details of associated medical and psychiatric conditions.^1^, ^9^ Manifestation of coin pica is diverse, and therefore, its precise diagnosis is sometimes difficult or missed unless the patient admits to swallowing foreign bodies or it is revealed in imaging procedures.^10^

**Figure 2:**
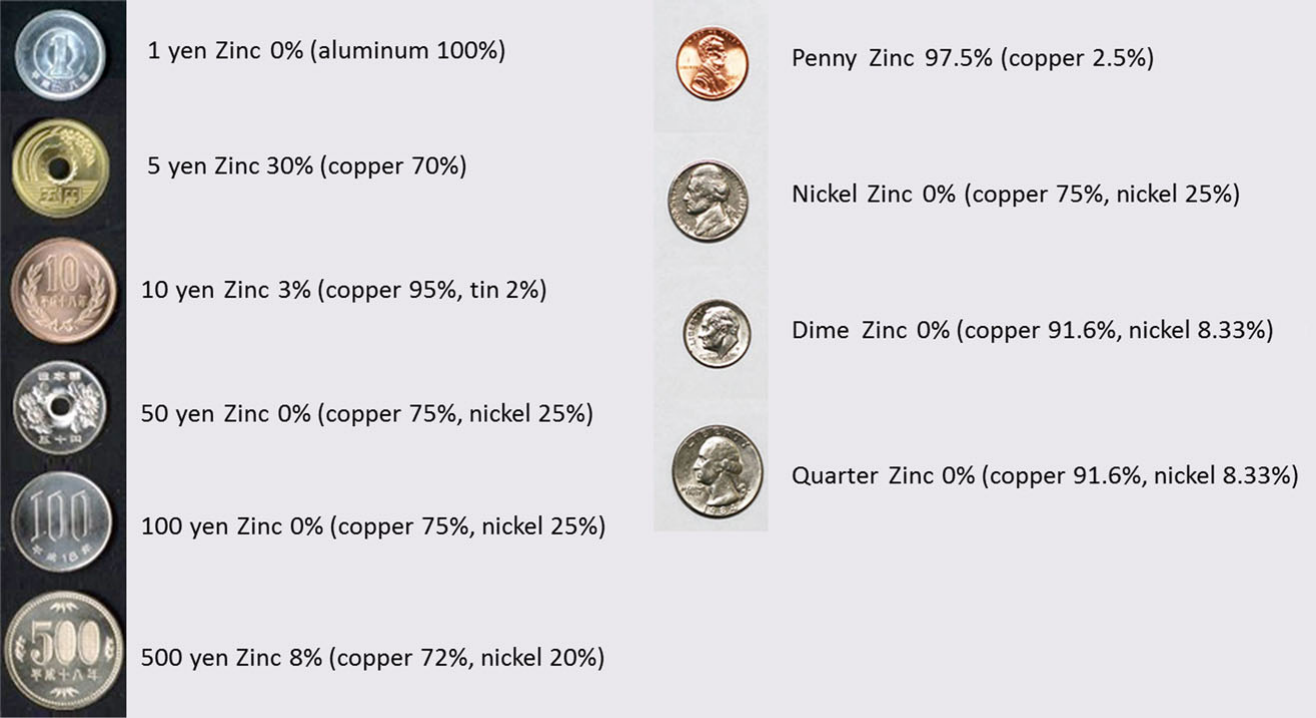
Japanese and American coins and their metal composition.

As far as we know, there have been only six case reports of coin pica between 1975 and 2017 on PubMed (Table 2).^2^, ^3^, ^4^, ^5^, ^6^, ^7^ Psychiatric history was characteristic of these coin pica cases. The main complication in these cases was metal intoxication; one case showed copper toxicity and five cases showed zinc toxicities. Severe metal intoxication can result in multiple organ failure and mortality. The total number of removed coins in our case was 1,894 coins, which weighed approximately 8 kg, exceeding previous cases with a maximum of 600 coins. We analyzed the composition of the ingested coins. The metal composition of Japanese coins and American coins are shown in Fig. 2. Although most of the coins removed were 10‐yens, which mainly consist of 95% copper and 3% zinc, the patient did not show any significant signs of metal toxicity, which was supported by the normal serum concentrations. Copper toxicity includes liver dysfunction, kidney dysfunction, and severe anemia, none of which the patient showed.^2^ D‐penicillamine and dimercaprol are generally the treatment of choice for copper toxicity. Furthermore, the coins removed were almost intact without any significant changes in corrosion or formation of bezoars. These facts suggest that the patient ingested the coins in a short period of time before seeking medical attention, although it is difficult to prove due to the patient's mental disorder.

**Table 2:** Review of articles of coin pica cases

Case	Age	Sex	Chief complaint	Psychiatric history	Number or weight of coins (type/s)	Metal toxicity	Main problem	Outcome	Author	Year
1	58	F	Dyspnea	Psychiatric disorder	275 (penny, nickel, dime, quarter)	Copper	Anemia	Died	Yelin^2^	1987
2	55	M	Epigastric pain	Schizophrenia	461 (penny)	Zinc	MOF	Died	Bennett^3^	1997
3	58	M	Fatigue, nausea	Schizophrenia	Unremoved (penny)	Zinc	Anemia	Survived	Hassan^4^	2000
4	54	M	Vomiting	Schizophrenia	1,870 g (penny)	Zinc	MOF	Died	Kumar^5^	2001
5	38	M	Epigastric pain	Schizophrenia	275 (penny)	Zinc	Anemia	Survived	Pawa^6^	2008
6	57	F	Lethargy	Schizophrenia	600 (penny)	Zinc	MOF	Survived	Dhawan^7^	2008
7	51	M	Fatigue	Schizophrenia	1,894 coins, 8,086 g (yen)	No toxicity	Gastric perforation	Survived	Sekiya^a^	2017

a Our case. F, female; M, male; MOF, multiple organ failure.

In addition, and in contrast to our case, none of the previous cases manifested gastrointestinal tract perforations, but did show metal intoxications and anemias (Table 2). We speculate that the perforation was due to erosion caused by pressure of the heavy metals on the gastric lumen and/or excessive distention of the stomach. In regards to the procedure, we determined there was no alternative option to removing the coins other than by laparotomy. Endoscopy and laparoscopy are less invasive procedures but unrealistic in this case due to the time it would have taken to remove the large number of coins. A wait‐and‐see strategy was inappropriate because the patient would not have survived without proper digestive function, even if there was no perforation.

As mentioned previously, the manifestation of pica is variable and could be asymptomatic. All physicians should be aware of the possibility of foreign body ingestion when seeing patients, especially those who have psychiatric histories and gastrointestinal symptoms, even if they are equivocal. Also, it is important to be aware that coin pica can cause metal toxicity as well as gastrointestinal symptoms. Appropriate examinations should be carried out when there is any suspicion of pica.

## Conclusion

Pica is common among patients with psychiatric disorders. A few cases regarding coin pica have been reported. However, ours is the first case report complicated by gastric perforation and documents the highest number of ingested coins ever reported.

## Disclosure

Informed consent: Written informed consent was properly obtained from the patient in accordance with the guideline of the ethical committee of Tokyo Medical and Dental University.

Conflict of interest: None.

## References

[1] Michalska A , Szejko N , Jakubczyk A *et al* Nonspecific eating disorders ‐ a subjective review. Psychiatr. Pol. 2016; 50: 497–507. 27556109 10.12740/PP/59217

[2] Yelin G , Taff ML , Sadowski GE . Copper toxicity following massive ingestion of coins. Am. J. Forensic Med. Pathol. 1987; 8: 78–85. 3578211 10.1097/00000433-198703000-00019

[3] Bennett DR , Baird CJ , Chan KM *et al* Zinc toxicity following massive coin ingestion. Am. J. Forensic Med. Pathol. 1997; 18: 148–53. 9185931 10.1097/00000433-199706000-00008

[4] Hassan HA , Netchvolodoff C , Raufman JP . Zinc‐induced copper deficiency in a coin swallower. Am. J. Gastroenterol. 2000; 95: 2975–7. 11051380 10.1111/j.1572-0241.2000.02336.x

[5] Kumar A , Jazieh AR . Case report of sideroblastic anemia caused by ingestion of coins. Am. J. Hematol. 2001; 66: 126–9. 11421292 10.1002/1096-8652(200102)66:2<126::AID-AJH1029>3.0.CO;2-J

[6] Pawa S , Khalifa AJ , Ehrinpreis MN *et al* Zinc toxicity from massive and prolonged coin ingestion in an adult. Am. J. Med. Sci. 2008; 336: 430–3. 19011402 10.1097/MAJ.0b013e31815f2c05

[7] Dhawan SS , Ryder KM , Pritchard E . Massive penny ingestion: the loot with local and systemic effects. J. Emerg. Med. 2008; 35: 33–7. 18180130 10.1016/j.jemermed.2007.11.023

[8] Syrakos T , Zacharakis E , Antonitsis P *et al* Surgical intervention for gastrointestinal foreign bodies in adults: a case series. Med Princ Pract. 2008; 17: 276–9. 18523393 10.1159/000129605

[9] Byard RW . A review of the forensic implications of pica. J. Forensic Sci. 2014; 59: 1413–6. 24975510 10.1111/1556-4029.12520

[10] Rose EA , Porcerelli JH , Neale AV . Pica: common but commonly missed. J. Am. Board Fam. Pract. 2000; 13: 353–8. 11001006

